# The relationship between lifestyle habits and obesity among students in the Eastern province of Saudi Arabia: using the Arab Teens Lifestyle (ATLS) questionnaire

**DOI:** 10.1186/s12889-024-19353-5

**Published:** 2024-08-21

**Authors:** Alexander Woodman, Margaret Coffey, Anna-Mary Cooper-Ryan, Nizar Jaoua

**Affiliations:** 1 School of Health and Society, University of Salford, Salford, Greater Manchester, UK; 2 Department of Human and Digital Interface, Woosong University, Daejeon, Republic of Korea

**Keywords:** ATLS questionnaire, Nutrition, Physical activity, Sedentary behaviours, Overweight, Obesity, Students

## Abstract

**Background:**

The Arab Teens Lifestyle (ATLS) questionnaire was an initiative to assess the lifestyle habits influencing obesity rates in the Middle East and North Africa (MENA) region, including physical activity (PA) patterns, sedentary and eating behaviours. Since its implementation, the ATLS questionnaire has been used in several studies among different age groups and populations. This instrument has not previously been administered among the university students of the Eastern Province of Saudi Arabia, where the obesity rates are the highest in the country. This research was the first that aimed to identify lifestyle habits influencing the rates of obesity among 18-25-year-old university students in the Eastern Province of the Kingdom of Saudi Arabia (KSA) using the ATLS questionnaire.

**Methods:**

Quantitative cross-sectional research among *n* = 426 students of the Eastern Province of Saudi Arabia using the ATLS questionnaire.

**Results:**

Out of *n* = 426 participants, *n* = 200 (47%) were categorised (using body mass index) as normal weight; *n* = 113 (26.5%) were overweight, and *n* = 73 (17.1%) were obese. The findings showed that most of the nutritional, PA, and sedentary behavioural factors (e.g., screen time) in the questionnaire were not associated with obesity status amongst the participants. In the obese group, more of the males that consumed fruits, French fries, cakes, sweets and doughnuts more than three times per week were likely to be obese, which was not the case for females.

**Conclusion:**

The reported lifestyles of the students could potentially lead to long-term negative health effects, which is of concern given the rising rates of overweight, obesity, and obesity-related non-communicable diseases (NCDs) among the Kingdom’s adult and ageing population. Further studies are recommended to explore the knowledge, attitudes, and perceptions of Saudi students in the Eastern Province in relation to PA, sedentary behaviours, and dietary habits, along with their views on how these can be improved.

## Introduction

Over the last four decades, malnutrition and obesity have been transformed from a minor public health problem in developed countries to a major global health concern affecting both developed and developing countries [1–3]. The latest report by the World Obesity Atlas (2023) showed that 38% of the world’s population is either overweight or obese [4]. Similar to global trends, the burden of overweight, obesity and obesity-related NCDs in the countries of the Eastern-Mediterranean Region (EMR) has greatly increased over the last three decades, reaching epidemic [5]. The highest levels of obesity in 16 EMR countries are reported in Kuwait, Egypt, United Arab Emirate (UAE), Kingdom of Saudi Arabia (KSA), Jordan and the Kingdom of Bahrain, with obesity prevalence varying from 74 to 86% in females and from 69 to 77% in males [5–7].

With one of the youngest populations in the world, where 51% of the 33.4 million people are under 25 years of age, KSA has one of the highest obesity and overweight prevalence rates [8–10]. Considering obesity, the national survey (2021) of Saudi residents (*n* = 4,709) in the 13 administrative regions of Saudi Arabia found the overall prevalence to be 24.7% [11]. Moreover, in this study, as with a large body of international literature [5, 12, 13] obesity was significantly associated with NCDs such as type 2 diabetes, hypercholesterolemia, hypertension, lung diseases, rheumatoid arthritis, sleep apnoea, colon diseases and thyroid disorders. Females were more obese (25.5%) compared to of males (17.9%) [11]. Obesity rates in the KSA show regional variation, with the highest rates in the Eastern Province, also known as Ash Sharqiyah, (29%) and central regions (20.5%) and the lowest in the southern regions (8.9%) [9, 14]. While these data were reported in 2019, the more recent study by Althumiri et al. (2021) showed that the Eastern Province (29.4%) was still the most obese of the Kingdom, followed by Riyadh (26.9%), while the lowest was Baha (14.3%) [11].

The Eastern Province is the largest in the KSA by area and one of the most important regions as it is the most industrialised part of the Kingdom and the third-largest oil-producing region in the world [15]. The industrialisation and rapid urbanisation of the Eastern Province have resulted in distinct lifestyle changes of the population. Changes in lifestyles (i.e., unhealthy food consumption and reduced PA) appear to be related to the overall body weight of students in the Eastern Province; with those found to be overweight ranging from 11.7 to 20.5% and obese from 9.5 to 20.5% [16–18].

A number of studies have been conducted among students of the Eastern Province to explore the prevalence of obesity, as well as their food choices. For example, a cross-sectional study conducted in Dammam among *n* = 260 female students found that 35.7% had snacks as their meal, and 46.9% were eating while watching television; 82.7% consumed fast food one to six times/week, and 73.1% consumed soft drinks more than seven times/week [19]. However, despite these behaviours, 51.5% were of normal weight, 19.2% were underweight, and only 3.8% were obese [19]. In contrast, an earlier study by Sabra et al. (2007) among male university students (*n* = 159) in Dammam showed that the prevalence of overweight and obesity was 24.5% and 22.6%, respectively [20]. Epuru and Al Shammary (2014), in their study on nutrition knowledge and its impact on food choices among Saudi students (*n* = 100), reported that both genders had poor nutritional knowledge and awareness, which influenced their food choices and dietary patterns [21]. In their study, 26% of males and 32% of females believed they had adequate nutritional education, although while 70% indicated that fruits and vegetables are healthy, only 10% believed that dairy products are good for health [21]. More recently research among medical students (*n* = 562) at Dammam University it was found that while students recognised the importance of leading a healthy diet and being physically active, their behaviour did not concur with their attitudes [22] Regardless of academic standing and gender, there was a high percentage (91.3%) of poor nutritional behaviours among students (*n* = 562) (e.g., intake of fast food and soft drinks) and PA was not undertaken regularly in 65% of males and 80% of female students [22].

The Arab Teens Lifestyle Study (ATLS) was an initiative to assess the lifestyle habits influencing obesity rates in the Middle East and North Africa (MENA) region, including PA patterns, sedentary and eating behaviours [23]. Since its implementation, it has been used in several studies among different age groups and populations in the MENA region (see for example UAE, Bahrain, Jordan, Oman, Tunisia, Morocco), as well as Riyadh [24–27]. However, it has not previously been administered among students in the Eastern Province of Saudi Arabia, where the obesity rates are the highest in the country. This research has become the first that aimed to identify lifestyle habits influencing the rates of obesity among 18-25-year-old university students in the Eastern Province of the KSA using the ATLS questionnaire, thereby filling the existing knowledge gap.

## Methods

### Study design and instrumentation

Quantitative methods typically provide numerical descriptions and estimates of the size and distribution of effects and allow tests for statistical significance [28]. This approach emphasises objective measurements and statistical analysis of data collected through surveys, questionnaires, and polls, or by processing existing statistical data using computational methods. Questionnaires offer an objective means of collecting information about the knowledge, beliefs, attitudes, and behaviour of the target population about the purpose of the study [28–30]. Thus, this study applied quantitative cross-sectional research among *n* = 426 students of the Eastern Province of Saudi Arabia using the ATLS questionnaire (with kind permission from the author of the questionnaire Professor Hazzaa Al-Hazzaa).

### ATLS questionnaire

The ATLS questionnaire, designed by Al-Hazzaa & Musaiger, (2011), is made up of four parts:

- *Socio-demographic questions and body mass index (BMI) questions*
- *Physical Activity/Inactivity*
- *Sedentary Behaviours*
- *Dietary Habits*.

The questionnaire has been validated and shown to be highly reliable for its target population, with an Intra-Class Correlation (ICC) of 0.85 [23]. In addition, the ATLS questionnaire results were subjected to the Cronbach Alpha test to assess the internal consistency reliability of a set of test items. During the development study, the 42-item ATLS questionnaire yielded an acceptable Cronbach Alpha value of 0.7 [23]. All parts were used in the current study.

### Study sample and recruitment

This research used purposive sampling, that is, participants who meet the criteria to address the research aim, namely 18-25-years-old university students in the Eastern Province of Saudi Arabia. According to General Authority for Statistics (GASTAT), as of autumn 2020, the youth population of the Eastern Province numbered 694,269. Hence, the minimum sample size needed in the Eastern Province was determined so that the sample proportion would be within ± 0.05 of the population proportion with a 95% confidence level [23, 31]. The population proportion has been assumed to be 0.5, as this assumption yields the highest possible value for the required minimum sample size [23, 31], which ensures the sufficiency of the study sample. This value can be determined as follows:

$$\:\text{n}\:=\:{{z}_{\alpha\:/2}}^{2}\:\text{*}\text{p}\text{*}(1-\text{p})\:/\:{\text{E}}^{2}\:$$

where z_α/2_ ≈ 1.96 is the critical value of the Normal distribution that corresponds to the 95% level of confidence (i.e., α/2 = 2.5%; the right-tail area under the normal curve), E = 0.05 is the desired margin of error (sample proportion maximum deviation from population proportion), and *p* = 0.5 is the assumed population proportion.

Based on these indicators and previous studies performed in this region, *n* = 384 + participants were recruited. Considering the sample size of *n* = 384 + and reviewed standards on the responsiveness and representativeness, a response rate of ≥ 80% was targeted.

Seventeen higher educational institutions in the Eastern Province of KSA were contacted via e-letter, sent to the heads of the Universities, to determine whether they would be interested in contributing to the research. While three gave initial agreement to participate, only two of these higher educational institutions^1^ in the Eastern Province participated in the final data collection, i.e., 426 students.

### Data collection

The administrations of the two institutions agreed to collect the data at one place. In Saudi Arabia, email communication is mostly used by people working in administrative or management positions [32, 33]. Furthermore, according to the latest available data and the experience of researchers in communicating with Saudi students, although the younger generation of the Kingdom (i.e., students) have been found to use the internet for chatting and entertainment via social media, they are not sufficiently familiar with email services effectively [32, 33]. Therefore, considering the ongoing debates about web- or paper-based studies and taking into consideration the email culture in the KSA, the decision was made to conduct a paper-based questionnaire among the target population to achieve both adequate response rates and representativeness [32, 33]. The printed questionnaires and consent forms were placed in envelopes, sealed, and distributed among participants. Data collection took place in a lecture room in 10 sessions over five days, with a 4-hour break between sessions. The principal investigator (AW) and chaperone were present in the room to respond to any participant questions about the questionnaire. The number of participants did not exceed 45 plus the researcher, to allow for social distancing as per Ministry of Health (2020) recommendations. The ATLS was distributed in both English and Arabic, and students were free to choose how to complete the questionnaire.

BMI was determined based on the ratio of participants’ weight (in kilogrammes, kg) to the square of their height (in square metres, m2). The results were then converted into four BMI categories with the following cut-points for BMI: (1) < 18.5 kg/m2 underweight; (2) 18.50–24.99 kg/m2 normal weight, (3) 25.00–29.99 kg/m2 overweight; (4) 30.00 kg/m2 and above obese, as specified by the NIH Adult BMI Classification, developed by WHO [34]. Body weight was measured without shoes and with minimal clothing to 100 g using a calibrated portable scale (Seca 750, Medical Measuring Systems and Scales, Hamburg, Germany). To obtain meaningful values, the scale was checked so that the pointer of the scale read “0” by moving the adjusting wheel, following the Seca 750 scale manual. Height was measured to the nearest cm while the subject was in the full standing position (Frankfort horizontal plane) without shoes using a calibrated portable stadiometer.

### Procedures

Ethical approval was sought and obtained on 03/08/2020 from the Ethics Committee of the University of Salford (HSR1920-016). All participants were informed that their participation was voluntary, and refusal would not result in any sanctions, they were free to leave the study at any time without giving a reason, no part of their information could be traced back to them, and that all information provided would be treated as confidential.

### Data analysis

**Fig. 1:**
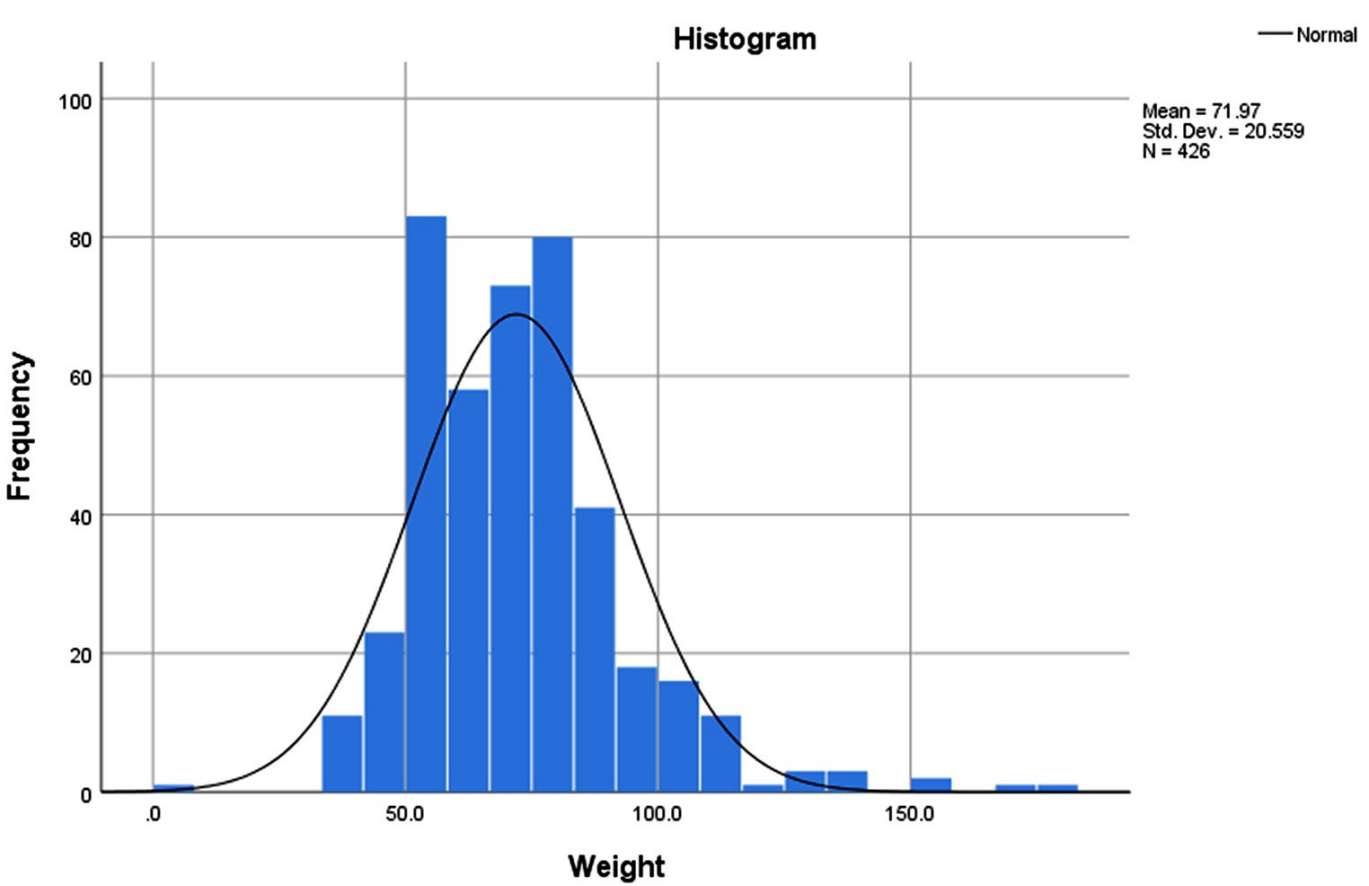
Weight frequency distribution for the study sample

Statistical analysis was performed using IBM Statistical Package for the Social Sciences version 25.0 (SPSS Inc., Illinois, USA). Sample description was given in frequencies and percent frequencies, along with a bar plot to visualize the sample pattern in terms of BMI profile. Normality of data was checked for participants’ height and weight, as these data were required for BMI calculation. It was found that the frequency distribution was significantly close to the normal distribution, as shown in Fig. 1 for the weight variable (Fig. 1).

Data was analysed following the ATLS questionnaire computation^2^. Stratified analysis was applied to investigate the association between sedentary/nutritional behaviours (explanatory variables) and obesity status (response variable) by adjusting for one of these factors. Odds ratios for obesity were determined using 95% confidence intervals between food frequency and screen time. For specific explanatory variables (potentially associated with obesity status), the median was used to split the sample in two groups, exposed vs. unexposed, which were presented in this order in the association tables (Tables 3 and 4, and 5). Chi-square tests were performed before and after stratification to test the significance of the association between sedentary/nutritional behaviours and the obesity status. The Breslow-Day test of homogeneity was used to determine whether the stratified odds ratios were significantly different, which would mean that the stratifying variable significantly modified the effect of the explanatory variable. At the 0.05 level of significance, the association/difference was considered statistically significant whenever the test provided a p-value not greater than 0.05.

## Results

### Part A: socio-demographic questions and body mass index (questions 1–7)

A total of *n* = 426 students (*n* = 133 females (31%), *n* = 293 males (69%)) took part in the survey (Table 1). The mean (M) ages of males and females were M = 22 (SD = 1.83) and M = 21 (SD = 1.73), respectively. Mean BMI for males was higher (M = 25.58 (SD = 6.57)) compared to females (M = 24.14 (SD = 6.27)) (Table 1).

**Table 1:** Demographic data and descriptive characteristics of study sample (*n* = 426)

Variable	Males *n* (%)	Females *n* (%)
**Age (year)**		
	30 (16.0) ± 0.9	32 (16.3) ± 1.2
18–20	69 (46.6)	79 (53.4)
21–23	166 (86.0)	27 (14.0)
24–25	58 (68.2)	27 (31.8)
Gender	293 (69)	133 (31)
Weight (kg)	76.70 = 20.50	61.57 = 16.51
Height (cm)	172.8 = 12.1	159.54 = 7.27
Body Mass Index	25.58 = 6.57	24.14 ± 6.27
**Marital Status**		
Divorced	1(100)	0
Married	15 (34.9)	28 (65.1)
Single	117 (30.9)	262 (69.1)
Others	0	3 (100)
**Children**		
1	2 (16.7)	10 (83.3)
2	3 (21.4)	11 (78.6)
3	4 (44.4)	5 (55.6)
More than 3	2 (100)	0
No children	122 (31.4)	267 (68.6)
**Nationality**		
Saudi	132 (32.1)	279 (67.9)
Non-Saudi	1 (6.7)	14 (93.3)

**Fig. 2:**
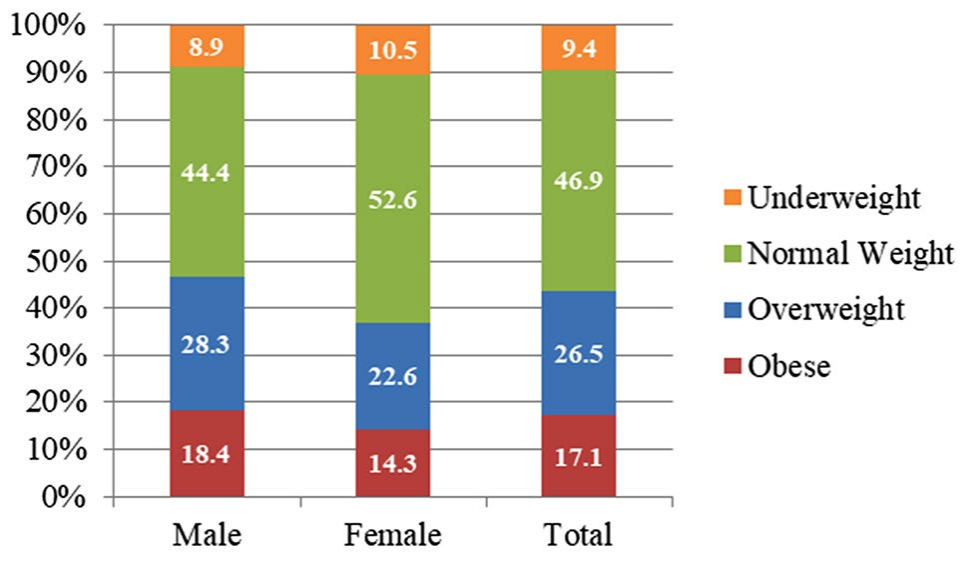
Distribution of BMI categories (*n* = 426)

Figure 2 shows that 47% of the sample were categorised as normal weight, 27% as overweight, 17% as obese, and 9% as underweight. Whereas the male BMI distribution was close to the sample distribution, among females, there were relatively more normal weight (53%), more underweight (11%), less overweight (23%), and less obese (14%).

### Part B: physical activity/inactivity (questions 8–34)

Part B of ATLS questionnaire explored the types of PA practiced by participants, considering frequency per week and minutes. The most frequent three types of PA were using the staircase *n* = 389 (91.5%), walking *n* = 320 (75%), and household work *n* = 261 (61.3) (Table 2). One of the challenges was that it was not possible to understand the detail about PA, i.e., how many minutes were spent undertaking given types of activity, as this question was not completed by most participants.

**Table 2:** ATLS - physical activity (*n* = 426)

Rank	Physical activity	Respondents for PA *n* (%)
1	Using staircase	389 (91.5)
2	Walking	320 (75)
3	Household work	261 (61.3)
4	Jogging/Running	242 (57)
5	Vigorous Intensity Sports	167 (39)
6	Moderate Intensity Sports	126 (30)
7	Strength training	123 (29)
8	Swimming	104 (24.4)
9	Cycling	88 (20.7)
10	Self-defence sports	48 (11.3)

### Part C: sedentary behaviours (questions 35–40)

Part C of the ATLS questionnaire explored sedentary behaviours, i.e., screen time (including watching TV, playing video games, computer, and social media) and their potential influence on the students’ weight. It was found that, in both groups related to screen time (i.e., those who spent more than two hours of screen time and those who spent less than two hours), the obesity rates were close to that of the entire sample. Spending more than two hours of screen time seemed to make no difference in odds for obesity (OR = 1.07), which was confirmed by Chi-square test (*p* > 0.05). When stratifying by gender, females who spent less time on screen were 1.3 (≈ 1/0.77) times more likely to be obese (15.2% vs. 12.2%), whereas in males, the screen time effect was almost the same as for the entire sample. However, the association was not significant for either males or females (p > 0.05). Adjusting for a nutritional factor potentially associated with obesity, namely, sugar-sweetened drinks intake, was also found to have no particular impact on this association, as the odds ratios were similar to that of the entire sample (Table 3).

**Table 3:** Analysis of screen time behaviours influencing the obesity status (*n* = 426)

Screen time (h/d)	Entire sample							
	Obese n (%)	Non-obese n (%)	Odds ratio (95% CI)	*p*-value				
≥ 2	31 (17.7)	144 (82.3)	1.07 (0.64–1.78)	0.7915				
< 2	42 (16.7)	209 (83.3)						
**Male**					**Female**			
	**Obese ** **n (%)**	**Non-obese ** **n (%)**	**Odds ratio ** **(95% CI)**	***p-value***	**Obese ** **n (%)**	**Non-obese ** **n (%)**	**Odds ratio ** **(95% CI)**	***p-value***
≥ 2	26 (19.4)	108 (80.6)	1.13 (0.62–2.03)	0.6933	5 (12.2)	36 (87.8)	0.77 (0.26–2.31)	0.6455
< 2	28 (17.6)	131 (82.4)			14 (15.2)	78 (84.8)		
**SSDI ≥ 2** ^**(a)**^					**SSDI < 2** ^**(b)**^			
	**Obese ** **n (%)**	**Non-obese ** **n (%)**	**Odds ratio ** **(95% CI)**	***p-value***	**Obese ** **n (%)**	**Non-obese ** **n (%)**	**Odds ratio ** **(95% CI)**	***p-value***
≥ 2	15 (18.8)	65 (81.2)	1.05 (0.51–2.14)	0.8975	16 (16.8)	79 (83.2)	1.13 (0.54–2.35)	0.7531
< 2	24 (18.0)	109 (82)			18 (15.3)	100 (84.7)		

(a) Sugar-sweetened drinks intake at least twice per week

(b) Sugar-sweetened drinks intake less than per week

### Part D: Dietary habits (questions 41–50)

Part D of the ATLS questionnaire explored dietary habits of the sample (using a scale of zero to a maximum intake of seven days per week). Fast food and vegetable intakes, potentially associated with BMI, were selected to analyse this association by stratification, using gender then another nutritional factor, namely, dairy products. Similarly to screen time, higher consumers of fast food had slightly greater odds for obesity (OR = 1.14) for both genders. However, females who consumed less fast food were 1.2 (OR= 1/0.89) more likely to be obese. In the fast food groups, females had lower rates (13.5%) of consuming fast food more than three times a week compared to males (19.5%), which was also true for consuming fast food less than three times a week, i.e., 14.8% females vs. 17.1% males. All these associations, before and after adjusting for gender, were not statistically significant (*p* > 0.05). When stratified by dairy (four or more dairy intakes per week), the effect of fast food seemed to change strongly, i.e., higher rates of dairy product consumption were associated with obesity among higher fast food consumers (24.8% vs. 18.1%). In contrast, with less than four intakes per week, the rate dropped to 9.5%. However, there was no modification among those who consumed fast food less than three times per week. As a result, the fast-food based odds ratio for obesity jumped from 1.14 among the whole sample to 1.68 among dairy product intake (DPI) ≥ 4 consumers, and down to 0.55 among those who consumed DPI < 4. Despite this effect modification, the association was still not statistically significantin sports and the daily practice (Table 4). However, a complementary test, namely a test of homogeneity, showed a significant difference between the stratified odds ratios (*p* = 0.04).

**Table 4:** Analysis of fast food intake effect on the obesity status (*n* = 426)

Fast food intake (t/w)	Entire sample							
	Obese n (%)	Non-obese n (%)	Odds ratio (95% CI)	*p*-value				
≥ 3	39 (18.1)	177 (80.9)	1.14 (0.69–1.89)	0.6095				
< 3	34 (16.2)	176 (83.8)						
**Male**					**Female**			
	**Obese ** **n (%)**	**Non-obese ** **n (%)**	**Odds ratio ** **(95% CI)**	***p-value***	**Obese ** **n (%)**	**Non-obese ** **n (%)**	**Odds ratio ** **(95% CI)**	***p-value***
≥ 3	32 (19.5)	132 (80.5)	1.18 (0.65–2.15)	0.5901	7 (13.5)	45 (86.5)	0.89 (0.33–2.44)	0.8277
< 3	22 (17.1)	107 (82.9)			12 (14.8)	69 (85.2)		
**DPI ≥ 4** ^**(a)**^					**DPI < 4** ^**(b)**^			
	**Obese ** **n (%)**	**Non-obese ** **n (%)**	**Odds ratio ** **(95% CI)**	***p-value***	**Obese ** **n (%)**	**Non-obese ** **n (%)**	**Odds ratio ** **(95% CI)**	***p-value***
≥ 3	30 (24.8)	91 (75.2)	1.68 (0.88–3.23)	0.1148	9 (9.5)	86 (90.5)	0.55 (0.23–1.31)	0.1730
< 3	18 (16.4)	92 (83.6)			16 (16.0)	84 (84.0)		

(a) Dairy products intake at least four times per week

(b) Dairy products intake less than four times per week

The association between vegetable consumption and obesity appeared to take a different shape. Thus, even before stratification, obesity rates were higher in participants who consumed vegetables less than three times per week (19.5%) compared to participants who consumed vegetables more than three times per week (16%). Those who consumed vegetables less than three times a week were 1.27 times more likely to be obese than those who consumed vegetables more than three times a week. This gap almost disappeared in males (close to 18% for both groups) and largely increased in females (23.1% vs. 10.6%), where those with lower vegetables intake had 2.5 times greater odds for obesity. A similar effect modification was found when adjusting for consumption of cakes/doughnuts/biscuits. Indeed, whereas the obesity rate was close to 16% in both vegetable-related groups with less than two intakes of cakes per week, there was a gap between the two groups with higher cake intake (23% vs. 16%). More precisely, those who consumed fewer vegetables were 2.57 times more likely to be obese, although the association between vegetable intake frequency and obesity status was not statistically significant (*p* > 0.05) before and after adjusting for stratifying variables, even though it was nearly significant among females (*p* = 0.06) (Table 5). In addition, the test of homogeneity did not show a significant difference between the stratified odds ratios (*p* > 0.05), which means that the effect of vegetable intake on obesity status was nearly insensitive to gender and cake intake. In this case, a weighted average of stratified odds ratios, called Mantel-Haenzel adjusted odds ratio, OR ≈ 1.3 would suggest that, after stratification by either gender or cakes intake, those consuming vegetables less than three times per week were about 1.3 times more likely to be obese.

**Table 5:** Analysis of vegetables intake effect on the obesity status (*n* = 426)

Vegetables intake (t/w)	Entire sample							
	Obese n (%)	Non-obese n (%)	Odds ratio (95% CI)	*p-value*				
< 3	26 (19.5)	107 (80.5)	1.27 (0.75–2.16)	0.3733				
≥ 3	47 (16.0)	246 (84.0)						
**Male**					**Female**			
	**Obese ** **n (%)**	**Non-obese ** **n (%)**	**Odds ratio ** **(95% CI)**	***p-value***	**Obese ** **n (%)**	**Non-obese ** **n (%)**	**Odds ratio ** **(95% CI)**	***p-value***
< 3	17 (18.1)	77 (88.9)	0.97 (0.51–1.82)	0.9166	9 (23.1)	30 (76.9)	2.52 (0.93–6.80)	0.0620
≥ 3	37 (18.6)	162 (81.4)			10 (10.6)	84 (89.4)		
**CDBI ≥ 2** ^**(a)**^					**CDBI < 2** ^**(b)**^			
	**Obese ** **n (%)**	**Non-obese ** **n (%)**	**Odds ratio ** **(95% CI)**	***p-value***	**Obese ** **n (%)**	**Non-obese ** **n (%)**	**Odds ratio ** **(95% CI)**	***p-value***
< 3	16 (22.9)	54 (77.1)	1.57 (0.79–3.12)	0.1995	10 (15.9)	53 (84.1)	0.97 (0.42–2.24)	0.9492
≥ 3	28 (15.9)	148 (84.1)			19 (16.2)	98 (83.8)		

(a) Cake/doughnut/biscuit intake at least twice per week

(b) Cake/doughnut/biscuit intake less than twice per week

## Discussion

This research was the first to identify nutrition and behavioural factors influencing the rates of obesity among 18-25-year-old university students in the Eastern Province of the KSA using the ATLS questionnaire. Despite established patterns of the highest rates of obesity in the Eastern Province (29.4%) of KSA [11, 35, 36], findings from this study, using the ATLS questionnaire showed that of *n* = 426 participants, almost half were of normal weight (47%) and only 17.1% were obese. Further analysis revealed a number of novel patterns that to some extent contradicted established ideas about factors associated with obesity. These patterns form the basis for future studies and research and are discussed in comparison with existing literature below.

Part B of the ATLS questionnaire explored PA patterns influencing the rates of obesity among 18-25-year-old university students in the Eastern Province of the KSA. Due to missing data from most of participants, this research could not examine the association between a lack of PA and obesity. However, walking was found to be the 2nd most frequently practiced PA by the participants, *n* = 320 (75%), consistent with an earlier national study from 13 administrative districts in Saudi Arabia, showing that among 26,000 households walking accounted for 56.05% of PA [9]. Further analysis showed that the number of males being physically active was higher compared to females (although not statistically significant). The evidence from Pakistan found that women participants either do not practice or participate very little due to the limitations of socioeconomic factors, religious values and culture, Saudi society and the vision of women’s activities in various fields have changed greatly in recent decades. This transformation also includes female’s active participation in sports and the daily practice of PA [37, 38]. One way to explain gender differences in PA in the Saudi setting and worldwide, as suggested by previous researchers, is to consider the participation of males and females in daily and leisure activities, with women tending to engage in fewer leisure activities and exercise less intensively than men, which is a global pattern [39, 40]. Hence, further qualitative research on this topic is recommended to gain an in-depth understanding of attitudes, perceptions, and possible limitations for female Saudi students’ lower physical activity levels compared to males [9, 41].

Part C of the ATLS questionnaire explored sedentary behaviours and their potential influence on obesity among the sample. In this study, it was found that, among females, spending less than two hours of screen time per day was associated with greater odds for obesity, although this was not significant, even when adjusting for a nutritional factor such as sugar-sweetened drinks intake.  These findings seem to confirm previous studies, which have reported no associations between screen time/sedentary behaviour and obesity in both males and females [42–44]. Thus, further research may enrich the evidence on additional factors (e.g., climate, infrastructure, sociodemographic factors) that influence sedentary behaviour in both males and females and its association with obesity [45–48].

Part D of the ATLS questionnaire explored dietary habits influencing the rates of obesity among 18-25-year-old university students in the Eastern Province of the KSA. This study found no significant association between breakfast consumption and any of the weight categories of the target population. These findings concur with previous studies, which highlight that although previous research has linked breakfast consumption to improved overall nutrition, higher levels of PA, and overall quality of life, the relationship between breakfast intake and body weight are not yet well understood [49–53]. Therefore, further research is recommended to explore students’ breakfast quality and the factors influencing their choice to consume or skip this meal, which could include other socioeconomic (e.g., education, employment, family) and lifestyle factors such as smoking, sleep etc. [54–56]. In addition, this study found no significant association between obesity status and the frequency of specific foods consumption, including fast food. Further findings of this study showed that fast food consumption was high for the entire sample, and being male was associated with eating fast food more than three times per week. These patterns are consistent with studies among Italian and Romanian students, where being male was associated with more consumption of fast food [57, 58]. Similarly, Alassaf et al. (2021) in their study among students from Central Saudi Arabia reported that more than half of male (53.8%) reported eating fast food three or more times a week, while more than half females (58.8%) reported eating fast food one or twice a week [59]. This finding is consistent with those of Al-Qahtani & Sundogji (2016), Yücel (2020), and Althumiri et al., (2021) who found no significant association between the BMI of students and their fast food consumption [11, 60, 61]. However, in this research, when stratified by milk and dairy products (≥ 4 d/w vs. < 4 d/w), fast food effect on obesity status was strongly modified in those who consumed fast food more than three times per week, revealing in this group a much higher obesity rate among higher dairy products consumers (24.8% after adjusting vs. 18.1% before adjusting) and a much lower rate among those with lower dairy profile (9.5%). Stratified odds ratios for obesity were also found to differ significantly among participants who consumed more dairy products (OR = 1.68) compared to the consumption of other food groups (OR = 0.55), suggesting a positive association between dairy consumption products and obesity. A recent meta-analysis examining the associations between milk and dairy consumption frequency and BMI found no significant difference between milk or yoghurt and obesity levels or between total dairy and its products and obesity levels [62–64]. However, the literature suggests that it is important to distinguish between dairy subtypes as their nutritional profiles differ and may also be associated with obesity differently [62–64]. Since the ATLS questionnaire does not include questions related to specific dairy products, further research is required to explore the choices made by the students in terms of the types of milk and dairy products and their energetic values.

Further analysis explored the frequency of vegetable consumption and its association with obesity status. It showed that the association was not statistically significant, although nearly significant among females (p = 0.06). Indeed, consuming vegetables less than three times a week was associated with 2.5 times greater odds for obesity among females (23.1% vs. 10.6%, compared to about 18% for both groups in males). On the other hand, this low vegetable-intake profile also had 1.6 times greater odds for obesity among those consuming cakes (and similar items) two or more times a week (23% vs. 16%, compared to about 16% for both groups in lower cake-intake profile). However, the effect modification by gender or cake intake frequency was found to be not significant and an adjusted odds ratio was estimated at about 1.3 after either of these stratifications. Although it is suggested that generous consumption of fruits and vegetables may help in weight control as they are rich in fibre and water [65, 66], which provides a satiety effect, this study is consistent with previous findings in the literature [18, 60, 62], that found no clear associations between fruit and vegetable consumption and a healthy weight. Taking into consideration the evidence on the association of fruit and vegetable consumption with BMI, students’ attitudes and preferences regarding fruit and vegetable choice should be explored in future studies, which may add value and shed some light on the existing conflicting literature.

Similar to previous studies that used the ATLS questionnaire, this study raised a number of questions and hypotheses that require further exploration through qualitative research. These questions include exploring barriers and facilitators to physical activity, reasons for skipping or eating breakfast, and perceptions of specific food groups. One of the most appropriate approaches to enrich additional data may be qualitative. Thus, while the quantitative method in this study provides numerical descriptions and estimates of the size and distribution of effects and allows testing of statistical significance, qualitative research, often described as using a naturalistic interpretative approach, will allow for the study of the phenomena from the inside and take the perspectives of research participants as a starting point [67–69].

### Strengths and limitations

The current study has strengths and limitations that require further consideration. Among the strengths of this study is that this was the first study that administered the ATLS questionnaire among the university students of the Eastern Province of the KSA. This study collected a large amount of information related to variables of interest, providing a larger picture of student obesity in this region. It also revealed, through stratified analyses, an important modification of the effect of specific foods intake on the obesity status. Another strength of the current study is the use of a combination of a validated self-report questionnaire (ATLS), which will enrich and complement the obesity data within the ATLS research initiatives, which is an important contribution to public health in Saudi Arabia.

In terms of limitations of this research, while three of the seventeen institutions gave initial agreement to participate, only two participated in the final data collection. Future studies should consider the recruitment of more participants to provide better knowledge and a more representative picture. BMI was the only suitable measure for specifying weight groups due to cultural specificities and social distancing caused by the pandemic. However, due to the limitations of these measures, caution is advised when using BMI as the sole measure of obesity in small observational studies. The greater number of males compared to females may potentially have skewed the findings. In addition, participants mostly did not report the number of minutes they engaged in a particular PA, which was a major limitation, in particular, for examining PA effect on the obesity status as well as for stratifying the analysis by this variable.

## Conclusions

This research showed that specific nutritional and sedentary behavioural factors did not influence obesity rates of students in the Eastern Province of Saudi Arabia. However, the reported lifestyles of the students, could potentially lead to long-term negative health effects which is important, given the rising rates of overweight, obesity, and obesity-related NCDs among the Kingdom’s adult and ageing population. Therefore, further qualitative studies are recommended to explore the knowledge, attitudes, and perceptions of Saudi students in the Eastern Province in relation to PA, sedentary behaviours, and dietary habits, along with their views on how these can be improved.

## Data Availability

Additional data associated with this study are available from the corresponding author, [AW], upon reasonable request.

## Footnotes

1 For the purposes of this study, to maintain anonymity and in line with GDPR, the names of the institutions that agreed to participate were coded as 1 and 2.

2 Hazzaa Al-Hazzaa (2012). ATLS Analysis Guidance - https://lh-hsrc.pnu.edu.sa/wp-content/uploads/2018/11/ATLS-Questionnare-Computations-Analyses-Instruction.pdf.

## Abbreviations

ATLS: Arab Teens Lifestyle Study
PA: physical activity
MENA: Middle East and North Africa
EMR: Eastern-Mediterranean Region
UAE: United Arab Emirate
KSA: Kingdom of Saudi Arabia
ICC: Intra-Class Correlation
GASTAT: General Authority for Statistics
SPSS: Statistical Package for the Social Sciences
M: Mean
SD: Standard Deviation
BMI: Body Mass Index
NIH: National Institutes of Health
NCD: Non-communicable diseases
